# Effectiveness of mobile health for exercise promotion on cardiorespiratory fitness after a cancer diagnosis: A systematic review and meta‐analysis

**DOI:** 10.1002/cam4.7079

**Published:** 2024-09-09

**Authors:** Megan E. Gregory, Weidan Cao, Saurabh Rahurkar, Fadi Haroun, James C. Stock, Sanam M. Ghazi, Daniel Addison

**Affiliations:** ^1^ Department of Health Outcomes & Biomedical Informatics University of Florida Gainesville Florida USA; ^2^ Department of Biomedical Informatics, College of Medicine The Ohio State University Columbus Ohio USA; ^3^ Cardio‐Oncology Program, Division of Cardiovascular Medicine The Ohio State University College of Medicine Columbus Ohio USA; ^4^ Division of Cancer Prevention and Control, Department of Internal Medicine, College of Medicine The Ohio State University Columbus Ohio USA

**Keywords:** cancer survivorship, cardio‐oncology, cardiorespiratory fitness, mobile health applications

## Abstract

**Background:**

Cancer survivors are at greater risk for cardiovascular‐related mortality. Mobile health (mHealth) is an increasingly prevalent strategy for health promotion, but whether it consistently improves cardiorespiratory outcomes after a cancer diagnosis is unknown. We sought to determine the effectiveness of mHealth fitness/physical activity interventions on cardiorespiratory fitness outcomes among cancer patients and survivors.

**Methods:**

Leveraging MEDLINE/PubMed, Scopus, and ClinicalTrials.gov, we identified studies through May 2023. Included studies provided a quantitative evaluation of an mHealth intervention in a primary or secondary capacity on cardiorespiratory fitness (6‐minute walk test, VO_2_max, 3‐minute step test, or systolic blood pressure; or any mention of cardiac measure) and were meta‐analyzed (using a random effects model) if they were a randomized controlled trial with sufficient quantitative information. Four coders were involved in applying inclusion/exclusion criteria, coding using a standardized data extraction sheet, and assessing study quality, with each study coded by at least two.

**Results:**

Of 656 articles, nine (*n* = 392) met systematic review inclusion criteria (mean age range 19–62 years, 71.9% female, 60.9% breast cancer). Interventions included mobile apps (*k* = 6), smartwatches (*k* = 2), or a smartwatch plus a supplemental web/mobile/tablet app (*k* = 1); median duration of mHealth‐use was 12 weeks. Seven (*n* = 341) fit criteria for meta‐analysis. mHealth was associated with improved cardiorespiratory fitness (*d* = 0.33; 95% CI = 0.07–0.60) compared to a control group. Relationships remained after accounting for lipid‐based outcomes (*d* = 0.30; 95% CI = 0.03–0.56). There was no evidence for heterogeneity or publication‐bias.

**Conclusions:**

mHealth exercise interventions appear to be a viable strategy for improving cardiorespiratory fitness after a cancer diagnosis.

## 1 INTRODUCTION

Cardiovascular disease (CVD) has become an increasingly common limitation to effective cancer therapy.^1^, ^2^ Over the last two decades, there has been dramatic improvement in early cancer survival. However, concurrently, CVD has become increasingly prevalent among patients initially surviving cancer, with a reported incidence of up to 38%.^1^ Many cardiovascular events cause serious declines in cardiorespiratory fitness and lead to debility. Unfortunately, widely available strategies to consistently improve cardiorespiratory fitness and reduce limiting CVD are largely unavailable.

Nonetheless, the landscape of healthcare has witnessed notable transformations spurred by legislative reforms and technological advancements, particularly in the United States, over the past decade. Health information technology (HIT) applications have experienced an upsurge in utilization as a result. Among these applications, electronic health records (EHRs) have become ubiquitous, often complemented by the availability and growing popularity of mobile health (mHealth) applications (or apps). Emerging isolated studies have shown that health promotion apps, such as those that guide users through exercise regimens or encourage physical activity, may improve cardiovascular risk by improving cardiorespiratory fitness and risk factors.^3^, ^4^ Given the high burden of CVD seen after a cancer diagnosis, novel mHealth strategies could present a widely available and effective means for fitness optimization. Whether app‐based interventions consistently improve cardiorespiratory outcomes after a cancer diagnosis, and the magnitude of these effects, are unknown.

The purpose of this study was to conduct a systematic review and meta‐analysis of existing literature pertaining to the utilization and effects of mHealth technology on cardiorespiratory fitness in cancer patients (defined as people in active treatment) and survivors (defined as people who completed active treatment). By analyzing the available data, this research aims to shed light on the efficacy of mHealth interventions in this context and provide valuable insights into the potential benefits they may offer in optimizing cardiorespiratory health for these groups. Finally, findings from our study may also inform future research agenda that may facilitate development of strategies to manage cardiorespiratory fitness among cancer patients and survivors.

## 2 METHODS

### 2.1 Literature search and inclusion criteria

We followed a protocol consistent with the Preferred Reporting Items for Systematic Reviews and Meta‐Analysis (PRISMA) guidelines. Specifically, we searched the PubMed, Scopus, and ClinicalTrials.gov databases through May 1, 2023, for any articles that evaluated mHealth apps in the context of cardiorespiratory fitness among cancer patients or survivors. We did not restrict our study to a beginning date. In order to be inclusive, we also evaluated gray literature to ensure that we included all possible studies that aligned with our search strategy. Articles were identified using keywords related to mHealth, health promotion, cancer patients, survivors, and cardiorespiratory fitness (see Appendix S1 for terms). Further, we contacted authors via email to request missing data if needed. Inclusion criteria entailed: (1) Study design: randomized controlled trials (RCTs) for the meta‐analysis, and RCTs and observational studies or pre‐post studies without a control group (including feasibility/pilot studies) for the systematic review; (2) Participants: cancer patients and/or survivors; (3) Intervention: utilization of mobile health (mHealth) technologies for physical activity or fitness; (4) Outcome: cardiorespiratory fitness (including but not limited to the 6 Minute Walk Test, VO_2_ max, and heart rate outcomes). Additionally, only empirical studies were included in our review and thus excluded articles such as government reports, white papers, commentaries, opinions, perspectives, and policy briefs. This review was not registered.

### 2.2 Screening and data extraction

**FIGURE 1:**
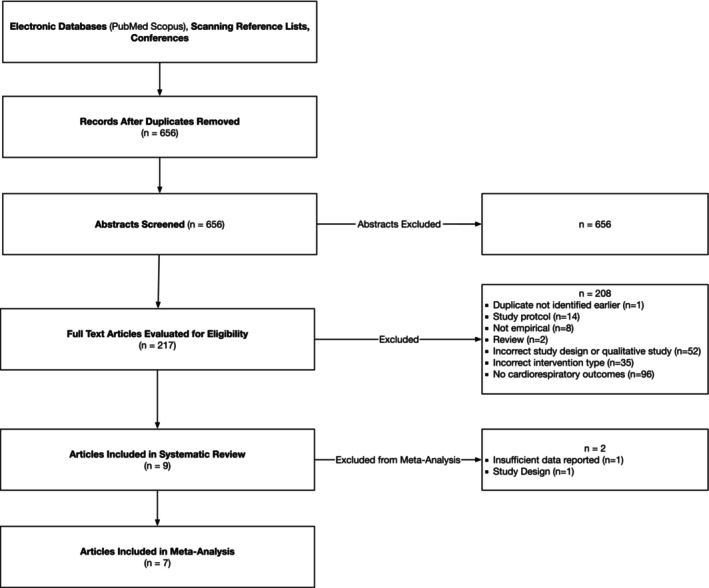
PRISMA diagram.

The keyword search identified 656 unique articles which were subjected to the process shown in Figure 1. Each article was independently reviewed by at least two reviewers with a total of four reviewers performing the initial title and abstract screening. Discrepancies were resolved through discussion and consensus. Data from included studies were extracted using a standardized data extraction form to systematically code relevant information, such as study characteristics, demographics, intervention details, outcome measures, and effect sizes. Cohen's d effect size index was used to quantify the effect size for articles in the meta‐analysis. When a negative change indicated a positive outcome (e.g., reduction in cholesterol), the inverse of the effect size was computed to ensure consistency in the direction of effect size interpretation. If multiple relevant outcomes were reported within a study, mean composites were created to obtain a single effect size per study. If a study reported multiple post‐intervention time points, data from the most proximal time point were used for the analysis. Four coders were involved in data extraction, with each study double‐coded by at least two reviewers. Discrepancies were resolved by the first author who reviewed the article in question and made a final decision.

### 2.3 Meta‐analysis

A random‐effects model was used to pool the effect sizes across studies, and studies were weighted by the reciprocal of the sampling variance using the DerSimonian and Laird method.^5^ The meta‐analysis was conducted using the “metafor” package in R.^6^ To assess publication bias, several methods were employed, including a funnel plot, the Trim and Fill method,^7^ the rank correlation test,^8^ Egger's test,^9^ and a cumulative meta‐analysis^10^ to explore the impact of study size on the overall effect size estimation. To assess the robustness and reliability of the meta‐analysis findings, sensitivity analyses, including influence analyses and leave‐one‐out analysis, were conducted. We also examined the impact of specific variables on the overall effect, including intervention duration (<12 weeks, >12 weeks), cancer type (breast cancer vs. others), and outcomes (6‐minute walk test only; inclusion vs. non‐inclusion of cholesterol outcomes).

### 2.4 Study quality assessment

We assessed quality of the included studies using two different tools: Cochrane Collaboration's Risk of Bias Tool^11^ (for RCTs) and a quality rating tool from the National Institutes of Health^12^ (for observational studies and pre‐post studies without a control group). Quality assessment coding was done by two independent coders, and discrepancies were resolved via discussion.

## 3 RESULTS

### 3.1 Study characteristics

Overall, from 656 articles, we identified nine articles (392 participants) that fit the systematic review inclusion criteria (see Table 1). Seven were RCTs with sufficient data reported to include in the meta‐analysis (see Figure 1 for PRISMA diagram). Most studies were of moderate‐to‐high quality overall (Tables 2 and 3). RCTs compared the intervention to another intervention (*k* = 4), usual care (*k* = 3), or a waitlist control group (*k* = 1). Interventions in studies in the systematic review included mobile apps (*k* = 7), smartwatches (*k* = 1), or a smartwatch plus an option to also use a web, mobile, or tablet app (*k* = 1). Supplements to these interventions included fitness trackers (*k* = 4), social media support groups (*k* = 3), a goal setting session (*k* = 1), and televideo exercises (*k* = 1). Intervention lengths ranged from 10 to 52 weeks, with 12 weeks being the most common (*k* = 5). Sample sizes ranged from 10 to 80 and focused on breast (*k* = 6), esophageal (*k* = 1), hematologic (*k* = 1), and multiple adolescent and young adult cancers (*k* = 1). Studies most commonly focused on survivors (*k* = 8), with two (*k* = 1) focusing on patients. None of the studies in this review included both survivors and patients. Studies were conducted in the United States (*k* = 5), Australia (*k* = 1), Japan (*k* = 1), Taiwan (*k* = 1), and China (*k* = 1). Among the studies conducted in the United States (*k* = 5, *n* = 166), 58% of the participants where White, 23% were Black, 5% were Asian, and 14% were of Other/Unknown race. All mHealth programs included exercise (*k* = 5), or physical activity (*k* = 4). Additional components included health education (*k* = 3), diet (*k* = 1), weight loss (*k* = 1), and smoking cessation (*k* = 1). The most common cardiorespiratory‐related outcomes were the 6‐minute walk test (*k* = 4) and VO_2_ max (*k* = 4). Other outcomes included the 3‐minute step test (*k* = 2), systolic blood pressure (*k* = 2), diastolic blood pressure (*k* = 2), total cholesterol (*k* = 1), HDL (*k* = 1), LDL (*k* = 1), resting heart rate (*k* = 1), and unspecified cardiovascular outcomes (*k* = 1).

**TABLE 1:** Study characteristics table.

Citation	Country	Cancer type	Cancer stage	Sample characteristics	Study design	Sample size	Intervention	Tool type	Intervention length	Time Since diagnosis, last treatment, or in remission	Between‐groups Δ Cohen's d	Included in Meta‐Analysis?
Chang et al., 2020^13^	Taiwan	Esophageal	Not reported	Survivors, Mean age: 56, Female: 9%	RCT vs. usual care	80 I: 40 C: 40	Exercise and health education	Smartwatch + web, mobile, or tablet app	12 weeks	Not reported	Mean of outcomes: 0.67 6MWT: 0.36 VO_2_ Max: 0.97	Yes (mean of outcomes used)
Chow et al., 2021^23^	USA (Washington)	Hematologic	N/A	Survivors, Mean age: 45, Female: 49%	RCT vs. another intervention	41 I: 24 C: 17	Physical activity, diet, smoking cessation	Goal setting session, fitness tracker, mobile apps	16 weeks	Average of 7.0 years (range 4.6–9.8) from last cancer treatment or transplant	Insufficient data reported; Reported there was no sig. between‐groups difference in change on cardiovascular outcomes	No (insufficient data reported)
Devine et al., 2020^24^	USA (New Jersey)	Multiple adolescent and young adult cancers	N/A	Survivors, Mean age: 19, Female: 49%	RCT vs. waitlist control	32 I: 12 C: 20	Exercise	Fitness tracker + mobile app	12 weeks	Intervention (M 9.46 years, SD 5.5), control (M 7.63 years, SD 4.4) since treatment completion	VO_2_ Max (Pre to 12 weeks): 0.43 No significant between groups improvement at 6 month delay	Yes (only scores from first time point used)
Dong et al., 2019^25^	China	Breast	I to III	Survivors (postoperative), Mean age: 50, Female: 100%	RCT vs. usual care	50 I: 26 C: 24	Exercise	Mobile app, fitness tracker, social media, and televideo	12 weeks	4 months to 2 years since completion of post‐operative radiotherapy/chemotherapy	VO_2_ max: 0.20	Yes
Ferrante et al., 2020^26^	USA (Multiple States)	Breast	0 to III	Survivors, Mean age: 62, Female: 100% African American	RCT vs. another intervention	35 I: 18 C: 17	Weight loss and physical activity	Fitness tracker + mobile app	12 weeks	Mean time from diagnosis was 6.60 years (SD 4.28)	6MWT: −0.27	Yes
Murphy et al., 2023^4^	Australia	Breast	Not reported	Patients, Mean age: 60, Female: 100%	RCT vs. usual care	80 I: 41 C: 39	Exercise	Mobile app	52 weeks	Not reported	Mean of outcomes: 0.42 6MWT: 0.51 SBP: 0.59 DBP: 0.15	Yes (mean of outcomes used)
Ochi et al., 2021^27^	Japan	Breast	I to IIa	Survivors, Mean age: 48, Female: 100%	RCT vs. another intervention	44 I: 21 C: 23	Exercise	Mobile app	12 weeks	Not reported	Mean of outcomes: 0.27 VO_2_ peak mL/kg/min: 0.47 VO_2_ peak L/min: 0.44 RHR: 0.90 SBP: −0.31 DBP: −0.06 6MWT: 0.20	Yes (only mL/kg/min used for VO_2_ max; mean computed among other outcomes)
Pope et al., 2018^28^	USA (Minnesota)	Breast	0 to III	Survivors, Mean age: 53, Female: 100%	RCT vs. another intervention	20 I: 12 C: 8	Physical activity and health education	Smartwatch + social media	10 weeks	Median time since diagnosis was >61 months	3MST: 0.10	Yes
Pope et al. 2019^29^	USA (Minnesota)	Breast	0 to III	Survivors, Mean age: 46, Female: 100%	Single arm pre‐post	10 I: 10 C: N/A	Physical activity and health education	Mobile app + social media	10 weeks	Mean time in remission was 34.5 months (SD 25.2)	N/A due to study design; No sig. pre‐post improvement in 3 min step test	No (due to study design)

**TABLE 2:** Study quality for RCTs.

Citation	Random sequence generation	Allocation concealment	Blinding of participants and personnel	Blinding of outcome assessment	Incomplete outcome data	Selective reporting	Other bias
Chang et al., 2020^13^	L	L	U	H	L	U	H
Chow et al., 2021^23^	U	L	L	L	U	L	H
Devine et al., 2020^24^	L	U	U	U	H	U	H
Dong et al., 2019^25^	L	L	H	L	H	U	H
Ferrante et al., 2020^26^	L	L	L	U	L	U	H
Murphy et al., 2023^4^	U	U	U	U	U	L	H
Ochi et al., 2021^27^	L	L	L	U	U	L	H
Pope et al., 2018^28^	L	H	H	H	H	U	H

Abbreviations: H, high risk of bias; L, low risk of bias, U, unclear risk of bias.

**TABLE 3:** Study quality for pre‐post studies.

	Objective clearly stated?	Eligibility criteria prespecified and clearly described?	Participants representative of clinical population of interest?	All eligible participants enrolled?	Sample size sufficiently large?	Intervention clearly described and delivered consistently?	Outcome measures prespecified, clearly defined, valid, reliable, and assessed consistently?	Outcome assessors blinded?	Loss to follow‐up 20% or less? Were those lost to follow‐up accounted for in analysis?	Examined changes in outcome measures pre‐post? *p* values for pre‐to‐post changes provided?	Outcome measures taken multiple times before and multiple times after the intervention?	If intervention conducted at a group level, did analysis account for use of individual‐level data?
Pope et al. 2019^29^	Yes	Yes	No	Yes	No	Yes	Yes	NR	No	No	No	NA

Abbreviations: NA, not applicable; NR, not reported.

### 3.2 Meta‐analytic results

**FIGURE 2:**
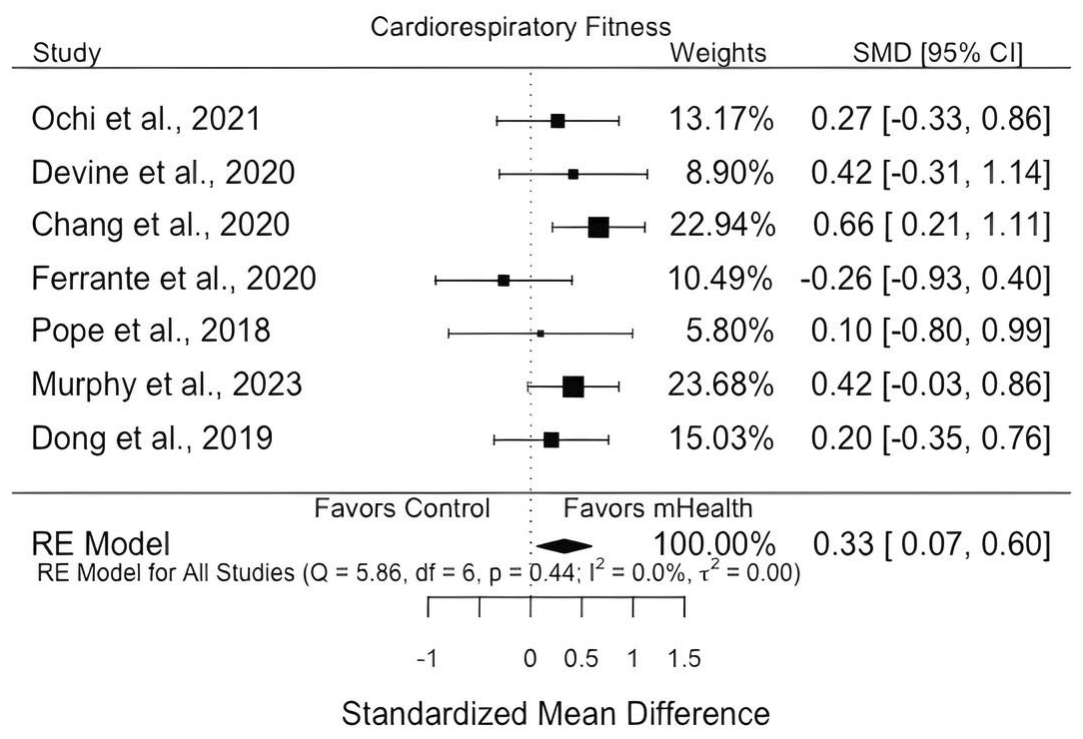
Forest plot of meta‐analysis on mHealth on cardiorespiratory fitness for cancer patients and survivors.

Across the seven studies in the meta‐analysis, mHealth was associated with improved cardiorespiratory outcomes (*d* = 0.33; 95% CI = 0.07, 0.60; see Figure 2 for forest plot). There was no evidence of heterogeneity (*Q* = 5.86, *p* = 0.44). There was no evidence of publication bias (details in Appendix S2). There was one possible outlier, Chang et al.,^13^ which if removed, would modify the overall effect size from *d* = 0.33 to *d* = 0.23 (Appendix S3). Upon further examination, we noted that this study differed from others in our sample in three ways that could potentially account for the differences: first, the cancer type was esophageal, whereas the other studies in the meta‐analysis were breast cancer (*k* = 5) or multiple adolescent and young adult cancers (*k* = 1); second, this was an esophagectomy‐based study, wherein patients had major surgery to remove the esophagus, which is unique in our sample of studies; third, the proportion of females in this study was low (9%), compared to moderate‐to‐high proportions of female participants (49%–100%) in the other studies in the meta‐analysis.

As sensitivity analyses, we ran the meta‐analysis in four other ways: adding in cholesterol outcomes from Murphy et al.,^4^ including only 6‐minute walk test outcomes, including only breast cancer studies, and including only studies with interventions of <12 weeks. Results maintained significance when cholesterol outcomes were added to the other cardiorespiratory outcomes (*d* = 0.30; 95% CI = 0.03, 0.56). Results of the meta‐analysis containing only the 6‐minute walk test (*k* = 4, *d* = 0.27, 95% CI = −0.20, 0.74), only breast cancer groups (*k* = 5, *d* = 0.20, 95% CI = −0.10, 0.51), and only studies <12 weeks (*k* = 6, *d* = 0.29, 95% CI = −0.06, 0.63) were not significant. Given the small number of studies, analyses were underpowered, and thus more studies should be conducted to confirm these findings. See Appendix S3 for more detail.

## 4 DISCUSSION

In this evaluation of the efficacy of mHealth‐based interventions, mHealth guided physical activity and exercise interventions were associated with increased cardiorespiratory fitness following a cancer diagnosis. These observations are of particular importance given the increasing observation of limiting cardiovascular disease after cancer treatment, and the relative paucity of available strategies to consistently improve outcomes. The observed efficacy of mHealth‐based exercise in improving cardiorespiratory outcomes adds to a growing body of evidence linking isolated electronic based applications with better cardiovascular measures. In animal studies, exercise attenuates chemotherapy‐induced cardiotoxicity, reflected by better ventricular performance, less subclinical injury, and fewer heart failure events. Similarly, in cancer and non‐cancer patients, mHealth based‐exercise strategies are linked with improved well‐being. In available isolated clinical studies, mHealth‐based applications improve body mass index, weight, and waist circumference from using mHealth in cancer survivors.^14^ Furthermore, in patients treated with systemic anticancer therapies, the use of an mHealth‐based strategy for improved pulmonary rehabilitation was safe, well‐accepted, and efficacious in improving functional outcomes. Yet, consistent data on the effects of mHealth‐based strategies on cardiorespiratory measures among cancer patients or survivors has been largely unavailable.

To our knowledge, this systematic review and meta‐analysis is the first of its kind to examine the impact of mHealth interventions on objective measures of cardiorespiratory fitness in cancer patients and survivors. Our findings suggest that mHealth interventions are effective at improving cardiorespiratory fitness in this population, with most trials showing such results in <12 weeks of mHealth use. Given the demonstrated importance of fitness^15^ and the high rate of cardiovascular disease in cancer survivors,^1^ these findings support continued research of mHealth interventions in this population. These findings support the preponderance of mHealth evidence in the oncology literature, with meta‐analyses and systematic reviews demonstrating that mHealth tools can improve QOL^16^, ^17^ and pain and fatigue management.^18^, ^19^ In addition to studies with patient‐centered variables, studies examining objective measures of physical activity^20^ and frailty^21^ among cancer patients have demonstrated the efficacy of mHealth interventions. Our findings are similar to a prior review^14^ which found improvements in body mass index, weight, and waist circumference from using mHealth in this population. Our work extends these findings by showing that cardiorespiratory outcomes such as the 6MWT, blood pressure, VO_2_ max, resting heart rate, and cholesterol can also be significantly improved.

These findings outline a potential role for mHealth in improving outcomes among cancer survivors, particularly those with comorbid CVD. Given the significant incidence of CVD in cancer survivors,^1^ mHealth could represent an important adjunctive treatment modality to prevent cardiovascular complications following a cancer diagnosis by providing educational materials, facilitating individualized coaching, and improving adherence to cardiovascular exercise, as these elements were present in many successful interventions. An alternative mechanism by which mHealth improved physical fitness was by inclusion of wearable fitness tracking technology which likely increased adherence to exercise regimens. Prior studies have shown that smartwatch‐based interventions significantly increase exercise and daily step count,^22^ therefore it is possible that the inclusion of wearable devices was a key explanation for the observed improvements in cardiopulmonary fitness. An additional mechanism could be the inclusion of telehealth coaching in several studies.

Our study has the following limitations; most notably, sample size. Despite our best attempts at identifying all possible studies, only nine studies met final inclusion criteria for a total sample size of 392 patients, with only seven studies able to be included in the meta‐analysis. However, this is also a reflection of the sparse nature of literature in the emergent use of mHealth apps to manage cardiorespiratory fitness among patients with cancer. Further, due to the small sample sizes of the included studies, statistical power was limited which may potentially affect generalizability. Another limitation is the heterogeneity of populations selected, which included several types of cancer at variable stages. It is possible that mHealth interventions could have variable effects in different populations, or at certain stages of disease. More studies will be required to elucidate the effects of mHealth in the widely heterogenous cancer survivor populations. Similarly, due to the small number of studies available, we were unable to test whether certain features of mHealth (e.g., frequency of reminders, addition of coaching, etc.) were more effective than others. Future work should be done to better understand this.

## 5 CONCLUSION

mHealth exercise interventions appear to be associated with small‐to‐moderate improvements in cardiorespiratory fitness for cancer patients and survivors. Considering the promise these apps may present as suggested by our findings and the sparse number of studies identified by our literature, there is a need for more research that utilizes rigorous and robust study designs. Further, future studies should also focus on improving precision of the effect size estimate, and to understand conditions under which mHealth is most impactful.

## AUTHOR CONTRIBUTIONS

**Megan E. Gregory:** Conceptualization (equal); data curation (equal); formal analysis (equal); investigation (equal); methodology (equal); project administration (equal); software (equal); supervision (equal); writing – original draft (equal); writing – review and editing (equal). **Weidan Cao:** Conceptualization (equal); data curation (equal); formal analysis (equal); methodology (equal); writing – review and editing (equal). **Saurabh Rahurkar:** Conceptualization (equal); data curation (equal); investigation (equal); methodology (equal); writing – original draft (equal); writing – review and editing (equal). **James C. Stock:** Data curation (equal); investigation (equal); validation (equal); writing – original draft (equal); writing – review and editing (equal). **Sanam M. Ghazi:** Data curation (equal); investigation (equal); validation (equal); writing – original draft (equal); writing – review and editing (equal). **Daniel Addison:** Conceptualization (equal); data curation (equal); funding acquisition (equal); investigation (equal); project administration (equal); validation (equal); writing – original draft (equal); writing – review and editing (equal). **Fadi Haroun:** Data curation (equal); validation (equal).

## CONFLICT OF INTEREST STATEMENT

This work was supported in part by National Institutes of Health (NIH)/ National Cancer Institute (NCI) grants K23‐HL155890 and R01HL170038 (Dr. Addison). DA was also supported by a Robert Wood Johnson Foundation (Harold Amos)‐American Heart Association Program grant. All other authors have reported that they have no relationships relevant to the contents of this paper to disclose.

## Supporting information


Appendices S1–S3.

## Data Availability

The data that support the findings of this study are available from the corresponding author upon reasonable request.

## References

[1] Florido R , Daya NR , Ndumele CE , et al. Cardiovascular disease risk among cancer survivors: the atherosclerosis risk in communities (ARIC) study. J Am Coll Cardiol. 2022;80(1):22‐32. doi:10.1016/j.jacc.2022.04.042 35772913 PMC9638987

[2] Armenian SH , Xu L , Ky B , et al. Cardiovascular disease among survivors of adult‐onset cancer: a community‐based retrospective cohort study. J Clin Oncol Off J Am Soc Clin Oncol. 2016;34(10):1122‐1130. doi:10.1200/JCO.2015.64.0409 PMC7357493 26834065

[3] Verburg A , Selder JL , Schalij MJ , Schuuring MJ , Treskes RW . eHealth to improve patient outcome in rehabilitating myocardial infarction patients. Expert Rev Cardiovasc Ther. 2019;17(3):185‐192. doi:10.1080/14779072.2019.1580570 30732481

[4] Murphy AC , Farouque O , Koshy AN , et al. Randomized controlled trial of a smartphone‐based intervention to enhance 6‐minute walk distance during breast cancer treatment: the SMART‐BREAST trial. Circulation. 2023;147(7):614‐616. doi:10.1161/CIRCULATIONAHA.122.062946 36342665

[5] DerSimonian R , Laird N . Meta‐analysis in clinical trials. Control Clin Trials. 1986;7(3):177‐188. doi:10.1016/0197-2456(86)90046-2 3802833

[6] Viechtbauer W . Conducting meta‐analyses in *R* with the metafor package. J Stat Softw. 2010;36(3):1‐48. doi:10.18637/jss.v036.i03

[7] Duval S , Tweedie R . Trim and fill: a simple funnel‐plot‐based method of testing and adjusting for publication bias in meta‐analysis. Biometrics. 2000;56(2):455‐463. doi:10.1111/j.0006-341X.2000.00455.x 10877304

[8] Begg CB , Mazumdar M . Operating characteristics of a rank correlation test for publication bias. Biometrics. 1994;50(4):1088‐1101. doi:10.2307/2533446 7786990

[9] Egger M , Smith GD , Schneider M , Minder C . Bias in meta‐analysis detected by a simple, graphical test. BMJ. 1997;315(7109):629‐634. doi:10.1136/bmj.315.7109.629 9310563 PMC2127453

[10] Borenstein M , Hedges LV , Higgins JPT , Rothstein HR . Introduction to Meta‐Analysis. John Wiley & Sons; 2009.

[11] Higgins JPT , Altman DG , Gotzsche PC , et al. The Cochrane Collaboration's tool for assessing risk of bias in randomised trials. BMJ. 2011;343(oct18 2):d5928. doi:10.1136/bmj.d5928 22008217 PMC3196245

[12] National Institutes of Health . Study Quality Assessment Tools. 2021 Accessed March 10, 2023. https://www.nhlbi.nih.gov/health‐topics/study‐quality‐assessment‐tools

[13] Chang YL , Tsai YF , Hsu CL , Chao YK , Hsu CC , Lin KC . The effectiveness of a nurse‐led exercise and health education informatics program on exercise capacity and quality of life among cancer survivors after esophagectomy: a randomized controlled trial. Int J Nurs Stud. 2020;101:103418. doi:10.1016/j.ijnurstu.2019.103418 31670173

[14] Murphy AC , Koshy AN , Mousley J , et al. Efficacy of mobile health cardiovascular risk‐reduction strategies in cancer survivors. Eur J Prev Cardiol. 2021;28(7):e4‐e6. doi:10.1177/2047487320907548 34247227

[15] Schmid D , Leitzmann MF . Cardiorespiratory fitness as predictor of cancer mortality: a systematic review and meta‐analysis. Ann Oncol off J Eur Soc Med Oncol. 2015;26(2):272‐278. doi:10.1093/annonc/mdu250 25009011

[16] Hernandez Silva E , Lawler S , Langbecker D . The effectiveness of mHealth for self‐management in improving pain, psychological distress, fatigue, and sleep in cancer survivors: a systematic review. J Cancer Surviv Res Pract. 2019;13(1):97‐107. doi:10.1007/s11764-018-0730-8 30635865

[17] Seiler A , Klaas V , Tröster G , Fagundes CP . eHealth and mHealth interventions in the treatment of fatigued cancer survivors: a systematic review and meta‐analysis. Psychooncology. 2017;26(9):1239‐1253. doi:10.1002/pon.4489 28665554

[18] Khoo S , Mohbin N , Ansari P , Al‐Kitani M , Müller AM . mHealth interventions to address physical activity and sedentary behavior in cancer survivors: a systematic review. Int J Environ Res Public Health. 2021;18(11):5798. doi:10.3390/ijerph18115798 34071342 PMC8198944

[19] Kim Y , Seo J , An SY , Sinn DH , Hwang JH . Efficacy and safety of an mHealth app and wearable device in physical performance for patients with hepatocellular carcinoma: development and usability study. JMIR Mhealth Uhealth. 2020;8(3):e14435. doi:10.2196/14435 32159517 PMC7097723

[20] Yates JW , Chalmer B , McKegney FP . Evaluation of patients with advanced cancer using the Karnofsky performance status. Cancer. 1980;45(8):2220‐2224. doi:10.1002/1097-0142(19800415)45:8<2220::aid-cncr2820450835>3.0.co;2-q 7370963

[21] Swenerton KD , Legha SS , Smith T , et al. Prognostic factors in metastatic breast cancer treated with combination chemotherapy. Cancer Res. 1979;39(5):1552‐1562. 427797

[22] Ringeval M , Wagner G , Denford J , Paré G , Kitsiou S . Fitbit‐based interventions for healthy lifestyle outcomes: systematic review and meta‐analysis. J Med Internet Res. 2020;22(10):e23954. doi:10.2196/23954 33044175 PMC7589007

[23] Chow EJ , Doody DR , Di C , et al. Feasibility of a behavioral intervention using mobile health applications to reduce cardiovascular risk factors in cancer survivors: a pilot randomized controlled trial. J Cancer Surviv. 2021;15(4):554‐563. doi:10.1007/s11764-020-00949-w 33037989 PMC8035343

[24] Devine KA , Viola A , Levonyan‐Radloff K , et al. Feasibility of FitSurvivor: a technology‐enhanced group‐based fitness intervention for adolescent and young adult survivors of childhood cancer. Pediatr Blood Cancer. 2020;67(9):e28530. doi:10.1002/pbc.28530 32589339 PMC7674223

[25] Dong X , Yi X , Gao D , et al. The effects of the combined exercise intervention based on internet and social media software (CEIBISMS) on quality of life, muscle strength and cardiorespiratory capacity in Chinese postoperative breast cancer patients:a randomized controlled trial. Health Qual Life Outcomes. 2019;17(1):109. doi:10.1186/s12955-019-1183-0 31242926 PMC6595606

[26] Ferrante JM , Devine KA , Bator A , et al. Feasibility and potential efficacy of commercial mHealth/eHealth tools for weight loss in African American breast cancer survivors: pilot randomized controlled trial. Transl Behav Med. 2020;10(4):938‐948. doi:10.1093/tbm/iby124 30535101 PMC7543085

[27] Ochi E , Tsuji K , Narisawa T , et al. Cardiorespiratory fitness in breast cancer survivors: a randomised controlled trial of home‐based smartphone supported high intensity interval training. BMJ Support Palliat Care. 2022;12(1):33‐37. doi:10.1136/bmjspcare-2021-003141 PMC8862092 34389552

[28] Pope Z , Zeng N , Zhang R , Lee H , Gao Z . Effectiveness of combined smartwatch and social media intervention on breast cancer survivor health outcomes: a 10‐week pilot randomized trial. J Clin Med. 2018;7(6):140. doi:10.3390/jcm7060140 29880779 PMC6025572

[29] Pope Z , Lee JE , Zeng N , Lee HY , Gao Z . Feasibility of smartphone application and social media intervention on breast cancer survivors' health outcomes. Transl Behav Med. 2019;9(1):11‐22. doi:10.1093/tbm/iby002 29471477

