# Supplementary material for: Effectiveness of mobile health for exercise promotion on cardiorespiratory fitness after a cancer diagnosis: A systematic review and meta‐analysis
Source: Cancer Med. 2024 Sep 9;13(17):e7079. doi: 10.1002/cam4.7079 (PMC11381959; doi:10.1002/cam4.7079)
Supplement: Supplementary file 1 — Appendices S1–S3. [file CAM4-13-e7079-s001.docx]

**Appendix S1: Search Terms Used**

("cancer"[Text Word] OR "tumor"[Text Word] OR "neoplasm"[Text Word] OR "malignanc*"[Text Word])

AND

("cardiotoxicity"[Text Word] OR ("cardi*"[All Fields] *AND* "toxicity"[Text Word]) OR "cardio toxicity"[Text Word] OR "onco cardiology"[Text Word] OR "oncocardiology"[Text Word] OR ("cardi*"[All Fields] *AND* "onco*"[Text Word]) OR "survivor*"[Text Word])

AND

(("mhealth"[Text Word] OR "m health"[Text Word] OR "mobile health"[Text Word] OR "app"[Text Word] OR "technology"[Text Word] OR "informatics"[Text Word] OR "mobile app*"[Text Word] OR "smartphone"[Text Word] OR "smart phone"[Text Word]) *AND* ("intervention"[Text Word] OR "trial"[Text Word] OR "experiment"[Text Word] OR "pilot"[Text Word] OR "experience*"[Text Word] OR "program*"[Text Word]))

**Appendix S2: Publication Bias Analyses**


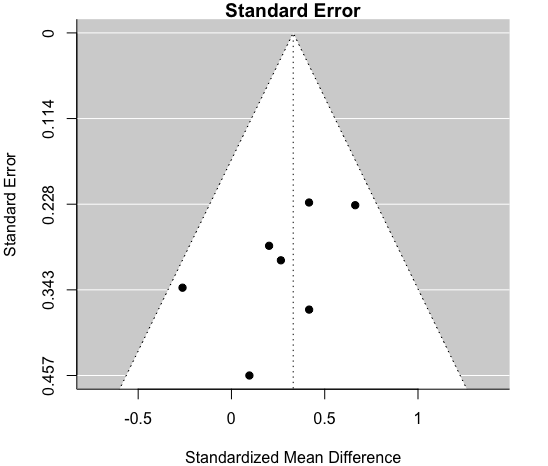


**Figure B1. Funnel plot for assessment of publication bias.**

**Table B1. Publication Bias Test Results**

| **Publication Bias Test** | **Result** |
| --- | --- |
| Rank correlation test | t = -0.43 (p =.24) |
| Egger’s test | *t*(5) = -1.75 (p = .14) |
|  |  |


**Table B3. Cumulative meta-analysis with a cut-point of n = 50.**

| Study | n | estimate | se | tval | pval | ci.lb | ci.ub | Q | Qp | tau2 | I2 | H2 |
| --- | --- | --- | --- | --- | --- | --- | --- | --- | --- | --- | --- | --- |
| Murphy | 80 | 0.42 | 0.23 | 1.84 | 0.07 | -0.03 | 0.86 | 0.00 | 1.00 | 0.00 | 0.00 | 1.00 |
| Chang | 80 | 0.54 | 0.12 | 4.34 | 0.14 | -1.04 | 2.11 | 0.59 | 0.44 | 0.00 | 0.00 | 1.00 |
| Dong | 50 | 0.46 | 0.13 | 3.58 | 0.07 | -0.09 | 1.00 | 1.65 | 0.44 | 0.00 | 0.00 | 1.00 |
| Ochi | 44 | 0.42 | 0.10 | 4.09 | 0.03 | 0.09 | 0.75 | 1.98 | 0.58 | 0.00 | 0.00 | 1.00 |
| Ferrante | 35 | 0.32 | 0.14 | 2.21 | 0.09 | -0.08 | 0.72 | 5.54 | 0.24 | 0.03 | 27.79 | 1.38 |
| Devine | 32 | 0.34 | 0.12 | 2.80 | 0.04 | 0.03 | 0.65 | 5.58 | 0.35 | 0.01 | 10.40 | 1.12 |
| Pope | 20 | 0.33 | 0.11 | 3.04 | 0.02 | 0.07 | 0.60 | 5.86 | 0.44 | 0.00 | 0.00 | 1.00 |

**Appendix S3: Outlier and Sensitivity Analyses**

**Table C1. Influence analysis.**

| Study | rstudent | dffits | Cook’s d | cov.r | tau2.del | QE.del | hat | weight | dfbs | Influence |
| --- | --- | --- | --- | --- | --- | --- | --- | --- | --- | --- |
| Ochi | -0.18 | 0.02 | 0.00 | 1.40 | 0.01 | 5.81 | 0.13 | 13.16 | 0.02 |  |
| Devine | 0.26 | 0.18 | 0.04 | 1.33 | 0.01 | 5.80 | 0.09 | 8.90 | 0.18 |  |
| Chang | 2.08 | 1.14 | 0.83 | 0.83 | 0.00 | 3.14 | 0.23 | 22.94 | 1.14 | *** |
| Ferrante | -2.64 | -0.90 | 0.41 | 0.56 | 0.00 | 2.45 | 0.10 | 10.49 | -0.90 |  |
| Pope | -0.50 | -0.07 | 0.00 | 1.23 | 0.01 | 5.58 | 0.06 | 5.80 | -0.06 |  |
| Murphy | 0.43 | 0.31 | 0.12 | 1.51 | 0.01 | 5.68 | 0.24 | 23.68 | 0.32 |  |
| Dong | -0.43 | -0.11 | 0.01 | 1.38 | 0.01 | 5.62 | 0.15 | 15.03 | -0.11 |  |

*Note.* *Potential significant influence.

**Table C2. Leave-one-out analysis.**

| Study | estimate | se | tval | pval | ci.lb | ci.ub | Q | Qp | tau2 | I2 | H2 |
| --- | --- | --- | --- | --- | --- | --- | --- | --- | --- | --- | --- |
| Ochi | 0.33 | 0.13 | 2.55 | 0.05 | 0.00 | 0.66 | 5.81 | 0.33 | 0.01 | 13.89 | 1.16 |
| Devine | 0.31 | 0.13 | 2.47 | 0.06 | -0.01 | 0.63 | 5.80 | 0.33 | 0.01 | 13.82 | 1.16 |
| Chang | 0.23 | 0.10 | 2.34 | 0.07 | -0.02 | 0.49 | 3.14 | 0.68 | 0.00 | 0.00 | 1.00 |
| Ferrante | 0.40 | 0.08 | 4.92 | 0.00 | 0.19 | 0.61 | 2.45 | 0.78 | 0.00 | 0.00 | 1.00 |
| Pope | 0.34 | 0.12 | 2.80 | 0.04 | 0.03 | 0.65 | 5.58 | 0.35 | 0.01 | 10.40 | 1.12 |
| Murphy | 0.29 | 0.13 | 2.19 | 0.08 | -0.05 | 0.64 | 5.68 | 0.34 | 0.01 | 11.91 | 1.14 |
| Dong | 0.34 | 0.13 | 2.69 | 0.04 | 0.01 | 0.67 | 5.62 | 0.35 | 0.01 | 10.98 | 1.12 |


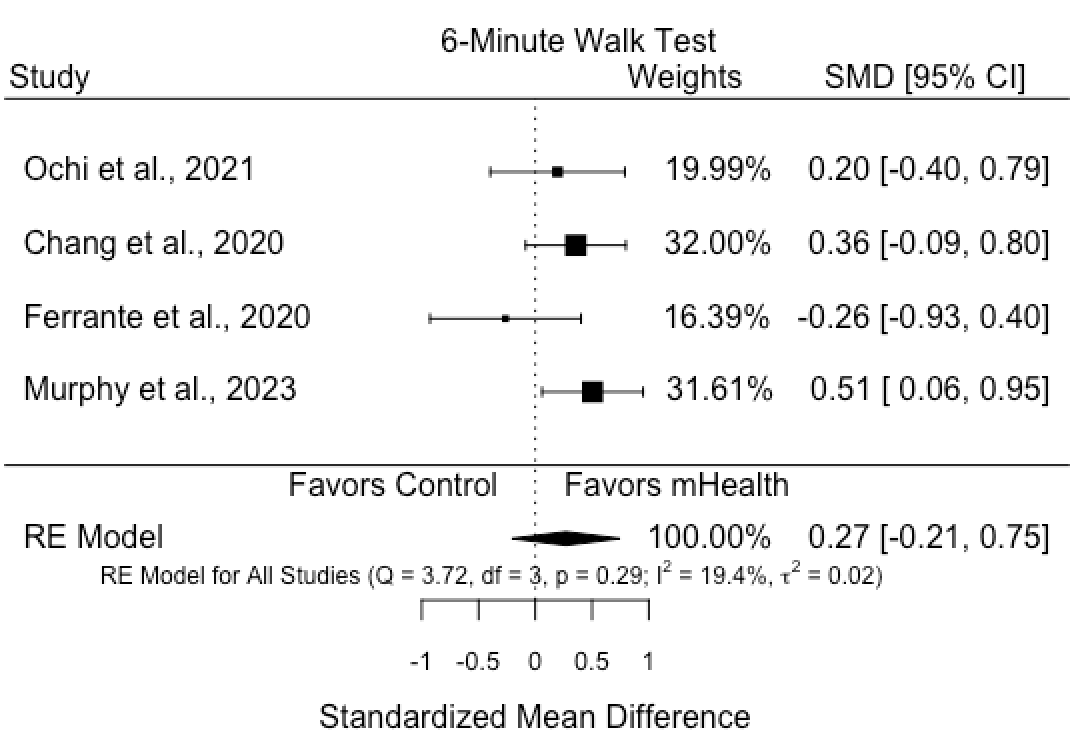


**Supplementary Figure 3. 6-minute walk test meta-analysis.**
